# A systematic simulation of the effect of salicylic acid on sphingolipid metabolism

**DOI:** 10.3389/fpls.2015.00186

**Published:** 2015-03-25

**Authors:** Chao Shi, Jian Yin, Zhe Liu, Jian-Xin Wu, Qi Zhao, Jian Ren, Nan Yao

**Affiliations:** ^1^State Key Laboratory of Biocontrol, Guangdong Key Laboratory of Plant Resources, Department of Biological Science and Technology, School of Life Sciences, Sun Yat-sen UniversityGuangzhou, China

**Keywords:** ceramides, salicylic acid, sphingolipid

## Abstract

The phytohormone salicylic acid (SA) affects plant development and defense responses. Recent studies revealed that SA also participates in the regulation of sphingolipid metabolism, but the details of this regulation remain to beexplored. Here, we use *in silico* Flux Balance Analysis (FBA) with published microarray data to construct a whole-cell simulation model, including 23 pathways, 259 reactions, and 172 metabolites, to predict the alterations in flux of major sphingolipid species after treatment with exogenous SA. This model predicts significant changes in fluxes of certain sphingolipid species after SA treatment, changes that likely trigger downstream physiological and phenotypic effects. To validate the simulation, we used ^15^N-labeled metabolic turnover analysis to measure sphingolipid contents and turnover rate in *Arabidopsis thaliana* seedlings treated with SA or the SA analog benzothiadiazole (BTH). The results show that both SA and BTH affect sphingolipid metabolism, altering the concentrations of certain species and also changing the optimal flux distribution and turnover rate of sphingolipids. Our strategy allows us to estimate sphingolipid fluxes on a short time scale and gives us a systemic view of the effect of SA on sphingolipid homeostasis.

## Introduction

Salicylic acid (SA), an important phenolic phytohormone, has well-known roles in pathogen-triggered defense responses including microbe-associated molecular pattern-triggered immunity, effector-triggered immunity, and systemic acquired resistance (Jones and Dangl, 2006; Spoel and Dong, 2012; Yan and Dong, 2014). SA also participates in abiotic stress responses (Vlot et al., 2009; Miura and Tada, 2014) and in plant development, including vegetative and reproductive growth (Vicente and Plasencia, 2011). SA also has indispensible functions in the maintenance of redox homeostasis (Durner and Klessig, 1995, 1996; Slaymaker et al., 2002) and respiratory pathways (Moore et al., 2002). The SA analog benzothiadiazole (BTH) activates the SA signaling pathway, triggers expression of defense genes (Shimono et al., 2007), and produces physiological effects similar to those produced by SA (Lawton et al., 1996).

As a key mediator of defense responses, the SA pathway affects many metabolic pathways. Sphingolipids are a family of complex lipids that have a serine-based head, a fatty acyl chain, and a long-chain base (LCB). Covalent modifications and variability in the length of the fatty acyl chain increase sphingolipid diversity. Sphingolipids are important structural and functional components of the plasma membrane (Hannun and Obeid, 2008) and have important functions in the plant immune response, abiotic stress responses,and developmental regulation (Chen et al., 2009; Pata et al., 2009; Markham et al., 2013; Bi et al., 2014). In *Arabidopsis*, ceramides, a group of sphingolipids, affect SA-mediated defense responses and programmed cell death (PCD). Some mutants in the sphingolipid metabolic pathway show high levels of expression of defense-related genes, accumulate SA, and undergo PCD. The ceramide kinase-deficient mutant *accelerated cell death 5* (*acd5*) accumulates SA and ceramides late in development, but shows increased susceptibility to pathogens (Greenberg et al., 2000; Liang et al., 2003; Bi et al., 2014). Wang et al. (2008) reported that the insertion knock-out mutant of *Arabidopsis* inositolphosphorylceramide synthase 2 (*erh1*) also spontaneously accumulates SA. Similar increases in SA levels have also been observed in the sphingosine transfer protein mutant *acd11* (Brodersen et al., 2002), the *Arabidopsis* sphingolipid fatty acid hydroxylase mutants *fah1 fah2* (König et al., 2012), and *mips1* (D-myo-inositol 3-phosphate synthase 1) mutants (Meng et al., 2009). Moreover, SA accumulation and PCD signaling mediated by MAPK affect the levels of free LCB (Saucedo-García et al., 2011). However, *fah1 fah2* mutants accumulate SA and have moderate levels of LCB (König et al., 2012). Thus, the SA and sphingolipid pathways have significant but complex crosstalk, particularly in defense and cell death.

Metabolic modeling performs well in prediction of physiological changes and metabolic outcomes resulting from genetic manipulation, where changes in metabolite levels have a strong effect on cellular behavior (Smith and Stitt, 2007; Stitt et al., 2010). The genome of *Arabidopsis thaliana* has been sequenced, making whole-genome metabolic reconstruction feasible (Thiele and Palsson, 2010; Seaver et al., 2012). Much of the early modeling work used steady-state Metabolic Flux Analysis (MFA), based on a steady-state model of the plant metabolic network, and on experiments using isotope labeling to trace metabolites of interest (Libourel and Shachar-Hill, 2008; Allen et al., 2009; Kruger et al., 2012). This method provided insights on metabolic organization and modes, but has difficulty in labeling heterotrophic tissues (Sweetlove and Ratcliffe, 2011), over-relies on manual curation of metabolic pathways (Masakapalli et al., 2010; Sweetlove and Ratcliffe, 2011; Kruger et al., 2012), and uses low-throughput detection, making systematic analysis difficult (Lonien and Schwender, 2009; Sweetlove and Ratcliffe, 2011).

By contrast, Flux Balance Analysis (FBA) overcomes many of the drawbacks of MFA. FBA establishes a model based on a group of ordinary differential equations that formulate a transient quasi-steady state of the metabolic fluxome of target pathways. The transient flux balance calculated by the FBA model has an almost-negligible duration compared to the long-term, fundamental metabolic changes that occur during development or in environmental responses (Varma and Palsson, 1994). In addition, FBA does not require isotopic labeling, suits a variety of trophic modes, and is more flexible than steady-state MFA in handling groups of flux distributions by linear programming and other methods for optimization under constraints (Edwards and Palsson, 2000; Reed and Palsson, 2003). Several *Arabidopsis* metabolic models based on FBA are available online (Poolman et al., 2009; Dal'Molin et al., 2010; Radrich et al., 2010).

Apart from FBA simulation, fluxomic changes can also be measured experimentally. To examine the response of sphingolipids to SA and BTH, we needed to determine and compare the turnover rates of sphingolipids. One of the major methods to measure turnover uses a time-course of stable isotopic incorporation into target metabolites, which are detected by mass spectrometry or nuclear magnetic resonance (Schwender, 2008; Hasunuma et al., 2010). The isotopic accumulation curve indicates the turnover of target metabolites.

Since metabolic changes substantially affect the crosstalk between SA and sphingolipids, in this study we constructed a metabolic model to simulate SA-related changes in the sphingolipid pathway. We constructed an *Arabidopsis* whole-cell FBA model including 23 pathways, 259 reactions, and 172 metabolites. Based on their relative enrichment and responsiveness to SA stimulation, our model includes 40 sphingolipid species, including LCBs, ceramides, hydroxyceramide, and glucosylceramides. Due to the lack of flux data on plant sphingolipid metabolism, we used ^15^N-labeled metabolic turnover analysis to measure sphingolipid flux in untreated plants and calibrate the FBA model. After the calibration, we also supplied the model with additional expression profiles from plants treated with SA and BTH. The FBA model was calculated *in silico* for prediction and comparison of the optimal flux distribution and flux variability in SA- and BTH-treated and untreated conditions. We then used metabolic turnover analysis with ^15^N-labeled samples to measure the flux changes directly. Both the computational model and the experiments showed consistent and significant changes in the sphingolipid pathway in response to SA and BTH. Our data gives us a systemic view of the effect of SA on sphingolipidhomeostasis.

## Materials and methods

### Plant materials

Wild type *Arabidopsis thaliana* ecotype Columbia seedlings were grown vertically on 1/2x Murashige and Skoog (MS) medium for 10 days after 2-day vernalization. The culture dishes were incubated at 22°C under a 16 h light/8 h dark cycle. For labeling the plant seedlings in liquid medium, the culture dishes were incubated at 22°C with 24 h light.

### Labeling and treatments

The different sphingolipids have many carbon atoms in different positions; therefore, labeling the only nitrogen in the serine-based head group provides an easier approach for LC-MS/MS measurements. We used ^15^N serine (Cambridge Isotope Laboratories, Inc. MA, USA) in the labeling experiment. Ten-day-old seedlings were transferred to N-deficient 1/2x MS liquid medium (Yoshimoto et al., 2004) in 12-well culture plates. 5 mM ^15^N-labeled serine was supplied to compensate for the shortage of nitrogen (Hirner et al., 2006) and used as the only source of isotope. For SA and BTH treatments, 100 μ M SA or 100 μ M BTH was supplied in the labeling medium. The seedlings were treated or not treated for 0, 1, 3, 5, 7, 9, and 24 h for ^15^N-labeled metabolic turnover analysis before sphingolipid extraction.

### Experimental measurement of turnover rate

Since serine has only one nitrogen atom and each sphingolipid has only one serine, the fraction of each labeled sphingolipid species can be measured as:

$${}^{15}\text{N fraction}\%={}^{15}{\text{N}}^{*}100/\text{N}$$

where ^15^N is the concentration of ^15^N-labeled molecules of a specific sphingolipid species, and N is the total concentration of that sphingolipid species, whether labeled or not.

The turnover rate of a sphingolipid species is calculated from the slope of the curve of the time-course of ^15^N incorporation from the initial time that the fraction begins to increase to the time that the fraction stabilizes. Also, the isotopic incorporation rate r can be calculated as:

$$\text{r}=\frac{{\text{d}}^{15}\text{Nfraction}}{\text{dt}}*\text{N}$$

In the measurement, the natural enrichment of ^15^N remains relatively constant between samples and treatments.

### Sphingolipid measurements

The plants cultured in labeling medium for the times described above were weighed and metabolically quenched by freezing in liquid nitrogen. Sphingolipid species were then extracted and measured by LC-MS/MS as described by Bi et al. (2014), with a slight modification to cope with isotopic-labeled sphingolipid species. Major sphingolipid species were subsequently analyzed with a Shimadzu 20A HPLC tandem AB SCIEX TripleTOF 5600^+^ mass spectrometer. The sphingolipid species were analyzed using the software Multiquant (AB SCIEX).

### Metabolic model construction

The Arabidopsis whole-cell metabolic model was constructed with 23 pathways, 259 reactions, and 172 metabolites. Primary metabolic pathways refer to the KEGG (Kyoto Encyclopedia of Genes and Genomes http://www.genome.jp/kegg/ Kanehisa et al., 2008), the AraCyc database (Mueller et al., 2003), and the AraGEM model (Dal'Molin et al., 2010), with manual curation for sphingolipid metabolism, including major ceramide, hydroxyceramide, and glucosylceramide species (Table S1). We used biomass as the objective function and the stoichiometries of major components were assigned to their biomass fraction, which comprises major carbohydrates, amino acids, and lipids, according to experiments or data provided in the literature (Fiehn et al., 2000; Welti et al., 2002; Dal'Molin et al., 2010). For sphingolipid species, the objective function stoichiometries were set to the adjusted isotopic incorporation rate in labeling experiments.

### Flux balance analysis (FBA)

Flux balance modeling uses a group of ordinary differential equations. The analysis requires a stoichiometric matrix (S) and a vector (v) built for each reaction, where s_ij_ in the S matrix is the stoichiometric number of the ith metabolite in the jth reaction and v_j_ is the rate of the jth reaction, which is subjected to upper and lower boundary constraints. To reach the *in silico* “quasi-steady state,” the following condition must be fulfilled:

S · v=0

After solving the FBA equation with the constraints above (Edwards and Palsson, 2000; Edwards et al., 2001), a linear-programming optimization method (Edwards and Palsson, 2000) was applied to pick the most plausible (groups of) flux distributions among the solution space according to the objective setting.

We applied isotopic incorporation rate as the reference for stoichiometry in the objective function. Considering that the stoichiometries of other components are biomass-derived (from AraGEM, Dal'Molin et al., 2010), we used optimization to find the proper fold-change of all isotopic incorporation rates simultaneously (**Table 2**, the column showing untreated isotopic incorporation rate) of sphingolipids, as their stoichiometries, to make a new model that deviated the least from the optimized steady-state flux distribution from the AraGEM model. Then, we optimized the individual stoichiometry of every sphingolipid species from the results of the first step to get a set of final stoichiometries (Table 1).

**Table 1:** **Overview of sphingolipid species in the FBA model**.

**Symbol**	**Sphingolipid species**	**Pool size (nmol· g ^−1^)**	**Stoichiometry in objective function**
d18:0 LCB	Long-chain base	0.2107728	0.050201
d18:1 LCB	Long-chain base	0.0404768	0.017119
t18:0 LCB	Long-chain base	0.280481	0.044619
t18:1 LCB	Long-chain base	0.1117734	8.05E-05
t18:1 c16:0	Long-chain ceramide	0.171892	0.14095
t18:0 c16:0	Long-chain ceramide	0.0097841	0.006289
d18:1 c16:0	Long-chain ceramide	0.0129473	0.017411
d18:0 c16:0	Long-chain ceramide	0.0404391	0.040446
t18:0 c24:0	Very-long-chain ceramide	2.1899963	0.47712
t18:1 c24:0	Very-long-chain ceramide	3.766825	0.775466
t18:0 c24:1	Very-long-chain ceramide	0.587771	0.119545
t18:1 c24:1	Very-long-chain ceramide	1.2656188	0.344293
t18:0 c26:0	Very-long-chain ceramide	0.7455185	0.129493
t18:1 c26:0	Very-long-chain ceramide	3.6843313	0.671015
t18:0 c26:1	Very-long-chain ceramide	0.0407943	0.005744
t18:1 c26:1	Very-long-chain ceramide	0.8207395	0.208064
t18:1 h160	Long-chain hydroxyceramide	0.8007893	0.154383
t18:0 h160	Long-chain hydroxyceramide	0.0852554	0.012748
d18:1 h16:0	Long-chain hydroxyceramide	0.0439154	0.020931
d18:0 h16:0	Long-chain hydroxyceramide	0.0365444	0.019623
t18:0 h24:0	Very-long-chain hydroxyceramide	1.2986488	0.01712
t18:1 h24:0	Very-long-chain hydroxyceramide	10.114958	1.148618
t18:0 h24:1	Very-long-chain hydroxyceramide	1.0769261	0.124845
t18:1 h24:1	Very-long-chain hydroxyceramide	0.0211909	1.53E-05
t18:0 h26:0	Very-long-chain hydroxyceramide	0.4134975	0.003149
t18:1 h26:0	Very-long-chain hydroxyceramide	2.2138763	0.218833
t18:0 h26:1	Very-long-chain hydroxyceramide	0.1257489	9.05E-05
t18:1 h26:1	Very-long-chain hydroxyceramide	1.268245	0.27478
t18:1 h16:0	Long-chain glucosylceramide	0.9171223	0.03589
t18:0 h16:0	Long-chain glucosylceramide	1.25E-06	9.00E-10
d18:1 h16:0	Long-chain glucosylceramide	2.908355	0.177984
d18:0 h16:0	Long-chain glucosylceramide	0.0239498	0.001506
t18:0 h24:0	Very-long-chain glucosylceramide	0.1940488	0.00014
t18:1 h24:0	Very-long-chain glucosylceramide	1.8239438	0.055296
t18:0 h24:1	Very-long-chain glucosylceramide	1.25E-06	9.00E-10
t18:1 h24:1	Very-long-chain glucosylceramide	2.1610275	0.057862
t18:0 h26:0	Very-long-chain glucosylceramide	1.25E-06	9.00E-10
t18:1 h26:0	Very-long-chain glucosylceramide	1.0588451	0.032563
t18:0 h26:1	Very-long-chain glucosylceramide	1.25E-06	9.00E-10
t18:1 h26:1	Very-long-chain glucosylceramide	0.7133198	0.016164

### *In silico* SA and BTH treatments

To incorporate the effect of exogenous SA and BTH on the wild-type plant into the model, we used published microarray data for SA- and BTH-treated *Arabidopsis* (for SA, van Leeuwen et al., 2007; for BTH, Wang et al., 2006). We assumed that the metabolic flux change followed the same trend as the respective gene expression levels. Therefore, we picked genes that changed more than 1.5-fold in SA-treated plants and more than 2-fold in BTH-treated plants (Table S2). Then, the adjusted model was recalculated for optimal flux distribution.

### Flux variability analysis (FVA)

The stoichiometry model is a self-balancing model in that any flux distributions that fulfill the constraints are involved in its solution space. Through the sampling of the solution space or sensitivity analysis, each reaction is tested for its possible upper flux limit and lower flux limit under constraints (Mahadevan and Schilling, 2003). The calculated range of each flux is an important indicator of the role of the corresponding reaction in the robustness of the whole network. To make a physiologically relevant estimation, we sampled the flux space that achieved at least 80% of the optimal objective rate (in our model, the biomass production) in untreated or treated conditions.

### Simulation environment

The model of *Arabidopsis* was built in SBML (Systems Biology Makeup Language) (Hucka et al., 2003) in XML format. SBML Toolbox 2.0.2 (Keating et al., 2006; Schmidt and Jirstrand, 2006) and COBRA Toolbox 2.0.5 (Schellenberger et al., 2011) in MATLAB 2012a (Mathworks Inc.; Natick, MA) were used for model construction and calculation. Linear programming was performed with GLPK (GNU Linear Programming Kit, http://www.gnu.org/software/glpk/). The rank-test and multiple covariance analysis were performed using IBM SPSS Statistics 19 (IBM Corp. Released 2010. IBM SPSS Statistics for Windows, Version 19.0. Armonk, NY: IBM Corp.).

## Results

### Model construction for plant sphingolipid metabolism

We used computational modeling and experiments to explore the changes in plant sphingolipid metabolism in response to SA. Although sphingolipids function as important components in plant development and stress responses, their metabolism remains obscure, with only a few network parameters that have been measured. FBA is well-suited to the simulation of a metabolic fluxome with poorly understood dynamics (Varma and Palsson, 1994), as optimization by FBA requires only the stoichiometric relationship in each reaction and the objective function. In our model, we obtained the numbers of molecules of reactants and products of known reactions from public databases (see Materials and Methods). For sphingolipid pathways (Table S1), we inferred the reactions that have not been determined from their atomic composition or similar reactions. Considering that metabolic balances are mainly affected by a few metabolites that are either in a hub of the network or have high turnover, we picked the sphingolipid species that are relatively abundant or central to the known network (Table 1). Since inositolphosphorylceramide and its derivatives are difficult to measure in plants, we excluded those species from our model.

### ^15^N-labeled metabolic turnover analysis of sphingolipids

To inform the objective function and to validate the model's prediction, we used the *in vivo* fluxomic method of ^15^N-labeled metabolic turnover analysis to directly measure the turnover rate of plant sphingolipids. In previous work, ^13^C was mostly used to examine the fluxome of central pathways such as glucose metabolism or photosynthesis (Hasunuma et al., 2010; Noack et al., 2010; Nöh and Wiechert, 2011), where limited numbers of labeled fragments are detected by mass spectrometry. However, the simplest sphingolipid has at least 18 carbon atoms, and their combined transitions, modifications, and fragmentation would generate large numbers of labeled fragments; therefore mass spectrometry quantification of ^13^C-labeled sphingolipid would be extremely difficult. To circumvent this difficulty, we used ^15^N, which will label only the single nitrogen atom in the head of each sphingolipid. To distinguish between artificial and natural ^15^N, we measured the composition of natural ^15^N sphingolipid in unlabeled samples, finding different levels of natural ^15^N in each sphingolipid species. This fraction is constant between measurements and treatments in each species, and thus cannot affect the comparison of isotopic incorporation rates between experiments.

**Figure 1:**
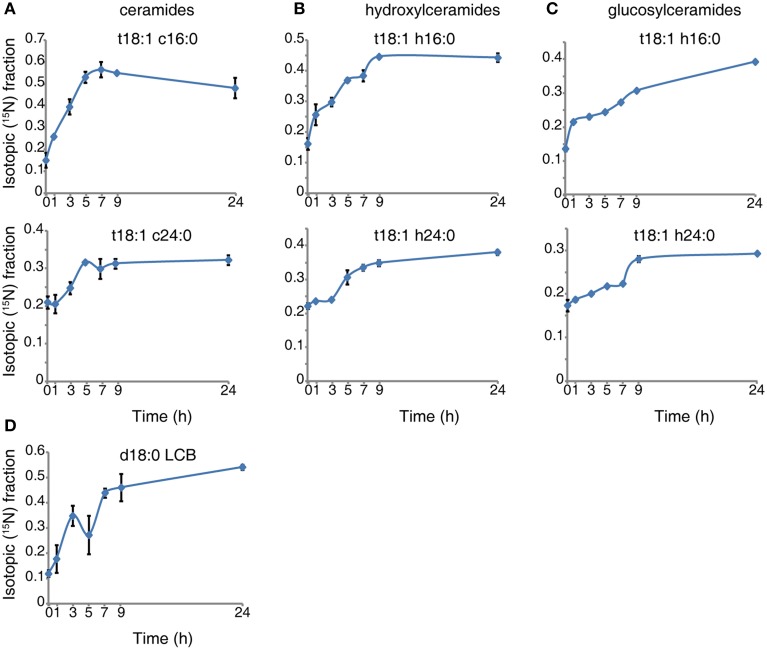
**^15^N incorporation curves for sphingolipid species**. Ten-day-old wild-type seedlings were transferred to 5 mM ^15^N- serine labeled N-deficient 1/2x MS liquid medium for the indicated times. Sphingolipids were then extracted and measured as described in Methods. The ^15^N fraction incorporation curve was calculated based on the formula shown in Methods. Error bars represent the means ± SE from triplicate biological repeats. The measured sphingolipid species were: ceramide **(A)**, hydroxyceramide **(B)**, gluocosylceramide **(C)** and LCB **(D)**. LCB and fatty acid in ceramide species represent, LCB; d/t (di/trihydroxy) 18 (18 carbon chain), 1 (one desaturation) followed by fatty acid; c/h/g (non-hydroxyl/hydroxyl/glucosy and hydroxyl) 24 (24 carbon chain), 0 (no desaturations).

We transiently labeled 10-day-old seedlings in a time course. The isotopic incorporation curves (see representative species shown in Figure 1) reveal that the labeled serine is absorbed and incorporated into sphingolipid in the first hour of labeling, and the sphingolipid then undergoes turnover at a uniform rate. For LCB (Figure 1D), ceramide (Figure 1A), and hydroxyceramide species (Figure 1B), the isotopic incorporation curves gradually flatten and finally reach a plateau of the isotopic fraction between 9 and 24 h. A noticeable, small drop occurs around the 5th hour of incorporation in LCB (Figure 1D). The incorporation of ^15^N in these simple sphingolipids is fast, and the final balanced isotopic fraction can reach 40–65% (Figures 1A,B,D). By contrast, for the glucosylceramides the labeled fraction rose constantly between 9 and 24 h (Figure 1C), and the glucosylceramides had a lower rate of incorporation than the ceramides or hydroxyceramides. Combined with the concentration of sphingolipids, we calculated the isotopic incorporation rate as shown inTable 2.

**Table 2:** **Isotopic incorporation rate for major sphingolipids, with or without 100 μ M SA or 100 μ M BTH treatments**.

**Symbol**	**Sphingolipid species**	**Isotope incorporation rate (nmol· g^−1^ ·h^−1^) untreated**	**Isotope incorporation rate (nmol· g^−1^ ·h^−1^) SA-treated**	**Isotope incorporation rate (nmol· g^−1^ ·h^−1^) BTH-treated**
d18:0 LCB	Long-chain base	0.062022	0.055779	0.038494^#^
d18:1 LCB	Long-chain base	0.005016	0.059469^*^	0.031829^*^
t18:0 LCB	Long-chain base	0.030297	0.049577	0.023784
t18:1 LCB	Long-chain base	1.43E-02	**8.94E-06^#^**	**5.44E-04^#^**
t18:1 c16:0	Long-chain ceramide	0.100845	0.241159^*^	0.221878^*^
d18:0 c16:0	Long-chain ceramide	0.04256	0.066754^*^	0.0477
t18:0 c24:0	Very-long-chain ceramide	0.386836	0.495358	0.505011^*^
t18:1 c24:0	Very-long-chain ceramide	0.418402	0.60068^*^	0.538219
t18:0 c24:1	Very-long-chain ceramide	0.217738	**0.144568^#^**	**0.176221**
t18:1 c24:1	Very-long-chain ceramide	0.485274	0.500902	0.547493
t18:0 c26:0	Very-long-chain ceramide	0.049354	0.048909	0.031827
t18:1 c26:0	Very-long-chain ceramide	0.136971	0.179349	0.184011^*^
t18:1 c26:1	Very-long-chain ceramide	3.44E-02	5.44E-02^*^	6.98E-02^*^
t18:1 h16:0	Long-chain hydroxyceramide	0.268339	0.253601	0.177361^#^
t18:1 h24:0	Very-long-chain hydroxyceramide	1.25246	1.139387	0.965043
t18:0 h24:1	Very-long-chain hydroxyceramide	0.092809	0.13231	0.167954^*^
t18:1 h26:0	Very-long-chain hydroxyceramide	0.157256	0.200213^*^	0.183134
t18:1 h26:1	Very-long-chain hydroxyceramide	1.86E-01	1.06E-01^*^	1.29E-01
d18:1 h16:0	Long-chain glucosylceramide	0.142007	0.126636	0.199323^*^
t18:1 h24:0	Very-long-chain glucosylceramide	0.076921	0.13433^*^	0.265554^*^
t18:1 h24:1	Very-long-chain glucosylceramide	0.073858	0.076487	0.15701^*^
t18:1 h26:0	Very-long-chain glucosylceramide	0.040668	0.053585	0.060641^*^

**Figure 2:**
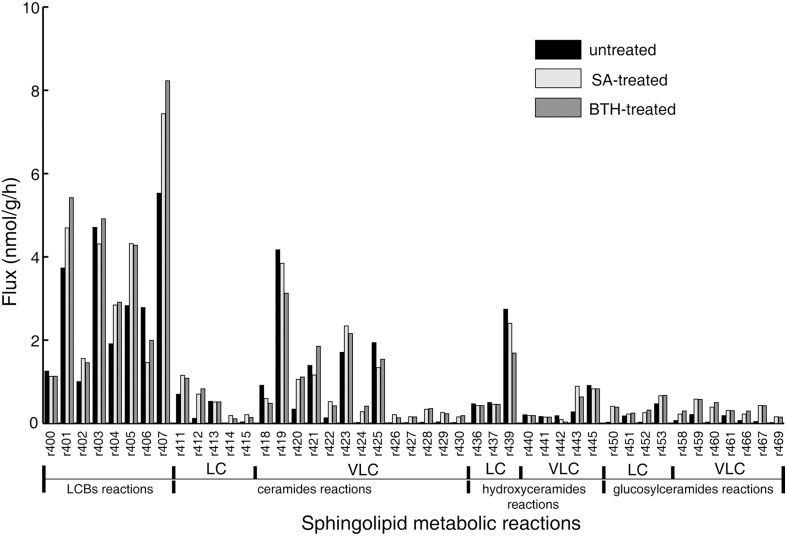
**Simulated flux distribution of selected sphingolipid species**. The untreated plants (black) and *in silico* SA (light gray) and BTH-treated plants (gray) were taken from the flux balance model. The effects of exogenous SA and BTH were simulated by changing the target flux bound proportional to its related gene expression alteration identified by published microarray data (Wang et al., 2006; van Leeuwen et al., 2007). LC, long-chain (≤C18); VLC: very-long-chain (>C18).

* and # indicate significant up and down, respectively (P < 0.05, FDR < 0.05 in multiple covariance analysis) of incorporation rate compared to untreated plants. The bold numbers are in disagreement with simulation data shown in Figure 2.

### Flux balance analysis (FBA) of the flux distribution in untreated plants

The objective function in the FBA model guides the flux determination by simulating a transient flux distribution. However, at each time point, biomass is the complex result of development throughout the organism's life, and hence cannot provide relevant information for setting the objective function in our model of the *Arabidopsis* seedling. Instead, we built and adjusted the objective function stoichiometries of the sphingolipid pathway from the isotopic incorporation rates in the labeling experiments (Table 1). Then, we performed flux balance optimization. Figure 2 shows the simulated flux distributions of sphingolipid species in untreated plants.

The simulation data in Figure 2 show that LCBs, very-long-chain ceramides, and hydroxyceramides compose the highest fraction of total flux. Combined with the rapid isotopic incorporation and high fraction of stabilized isotopic final levels of LCB, ceramides, and hydroxyceramides (Figure 1), the results demonstrate that LCBs, the sphingolipids that have the smallest pool size, also have the highest turnover among plant sphingolipids. Very-long-chain ceramides and hydroxyceramides are important not only for their hub position connecting glucosylceramides and sphingosine, but also because they carry a huge flux throughput in sphingolipid turnover and thus help maintain sphingolipid homeostasis. Both the simulation and experimental results indicate that these sphingolipid species are probably more responsive to disturbance, and thus are frequently used by pathogens to manipulate or interfere with host sphingolipid metabolism (Markham et al., 2011; Bi et al., 2014).

Although the glucosylceramides have much larger pool sizes (Table 1) than the ceramides, hydroxyceramides, or LCBs, they have smaller metabolic fluxes than their precursors (Figure 2). These results are validated by the slow but lasting incorporation of isotope into glucosylceramide pools (Figure 1C). The relatively slow turnover is in accordance with the function of glucosylceramides as membrane structural components, indicating a slow but continuous accumulation in the cell membrane during plant development. The accordance of simulation and experimental results also supports our choice of objective function stoichiometry setting, as the scale of simulated and measured sphingolipid metabolic flux distribution (Figure 2 and Table 2) is nearly unrelated to the distribution of sphingolipid biomass (Table 1).

### *In silico* SA and BTH treatments

The FBA model hypothesizes the quasi-steady state condition of the target network, and we assume that the sphingolipid pathway will reach at least a transient metabolic balance after SA treatment. Thus, we employed the previous model simulating the resting state to predict the effects of SA treatment. We first used data from microarray analysis of SA- and BTH-treated plants to simulate the effect of these treatments on sphingolipid flux. Sphingolipid-related genes were selected (see Method) from two microarrays (Table S2). *LAG 1 HOMOLOG 2* (*LOH2*), which encodes a ceramide synthase (Brandwagt et al., 2000; Ternes et al., 2011), showed the highest up-regulation after both SA and BTH treatments, and other genes *SPHINGOID BASE HYDROXYLASE 2* (*SBH2*), *FATTY ACID/SPHINGOLIPID DESATURASE* (*SLD*), *FATTY ACID HYDROXYLASE 2* (*FAH2*), *SPHINGOSINE-1-PHOSPHATE LYASE* (*AtDPL1*) also had different expression levels in the two treatments. The reactions regulated by the genes with altered transcript levels were then picked for incorporation into the model. The flux boundaries of these reactions were altered based on the gene expression, and the adjusted model was recalculated for flux balance analysis.

Compared with the model simulating the resting state, *in silico* SA and BTH treatments resulted in a nearly three-fold increase of predicted flux in long-chain ceramide species (Figure 2), as expected from the up-regulation of *LOH2* in the microarray data. In particular, simulated SA and BTH treatment both produced a significant rise in predicted metabolism of trihydroxy glucosylceramides. This increase was not specific to fatty acid species, which showed an increase in both trihydroxy long-chain and very-long-chain glucosylceramides (Figure 2). These results are consistent with the data from ^15^N-labeled metabolic turnover analysis (Table 2). Interestingly, the microarray data showed no significant changes in genes that directly catalyze the pathways in glucosylceramide metabolism, nor any related to glucosylceramide, in response to SA or BTH treatment (Table S2). Considering the down-regulation of *SBH2* under BTH treatment, we believe that the increase of glucosylceramide metabolism may mainly be induced by the upstream up-regulation of *LOH2*. Since the increase of the turnover rate was not linked to metabolite concentration, the predicted changes of glucosylceramides are almost negligible by typical quantitative LC-MS/MS measurement, but the increase in lipid renewal may have indispensible functions in the sensitivity of membrane-based cell signaling.

In this simulation, although some genes change differently in response to SA and BTH treatment (Table S2), SA and BTH have similar effects on sphingolipid metabolism. Our model also proposes a possible mechanism by which BTH affects the network under flux balance constraint without mimicking all the gene expression changes of its counterpart.

### ^15^N-labeled metabolic turnover measurement of the effect of SA and BTH

To confirm the predictions of the model, we directly measured the *in vivo* flux change in response to SA and BTH treatments. For SA and BTH treatments, the isotope incorporation rate significantly increased for certain sphingolipid species such as C16 and C26 ceramides (Table 2). These results are consistent with our FBA model (Figure 2). After SA and BTH treatments, turnover increased for seven out of twenty-two and ten out of twenty-two major sphingolipids, respectively. Also, turnover decreased for two out of twenty-two and three out of twenty-two major sphingolipids after SA and BTH treatments, respectively. We found that the few inconsistencies between *in silico* predictions (Figure 2) and experimental data (Table 2) mainly came from LCB and glucosylceramides. Given the low *in vivo* level of LCB and the high variability of LCB measurement, the inconsistency of LCB turnover could result from experimental error. Interestingly, we found discrepancies between the effect of BTH and SA on glucosylceramide turnover. For example, the isotope incorporation rate significantly increased for glucosylceramides after BTH treatments (Table 2), indicating that it may underlie different mechanisms in the responses to BTH and SA.

### Flux variability analysis

To examine the change in network rigidity in response to SA and BTH treatments, we estimated the accessible flux ranges of sphingolipid species *in silico*. To make a physiologically relevant estimation, we sampled the flux space that achieved at least 80% of the optimal objective rate (in our model, the biomass production) under untreated or treated conditions. We sorted the flux range into three types (Oberhardt et al., 2010): rigid flux (flux range near zero but with non-zero flux value), bounded flexible flux, and infinitely flexible flux (boundary spans from 0 or -1000 to 1000 in the model). In the fluxome of treated and untreated plants, LCB fluxes were infinitely flexible (showing a high capacity to tolerate disturbance), ceramide and glycosylceramide fluxes showed bounded flexibility, and hydroxyceramide fluxes were rigid (Table 3). The limited flux variability of most sphingolipids is consistent with stoichiometric modeling result in *S. cerevisiae* (Ozbayraktar and Ulgen, 2011). Similar to the isotopic incorporation experiments, we found disturbances of flux variability in ceramide and glucosylceramide metabolic fluxes after SA and BTH treatments, indicating that plant cells have the freedom to adjust their sphingolipid flux homeostasis during defense processes.

**Table 3:** **Simulated flux variability of sphingolipid-related reactions in untreated and SA-treated plants**.

**Reaction ID**	**Reaction property**	**Flux range of untreated plant (nmol/g/h)**	**Flux category**	**Flux range of *in silico* SA-treated plant (nmol/g/h)**	**Flux category**	**Flux range of *in silico* BTH-treated plant (nmol/g/h)**	**Flux category**
r400	LCB synthesis	0.6816942	BF	0.933054	BF	0.7323672	BF
r401	LCB hydroxylation	996.1271	IF	990.0517	IF	940.56259	IF
r402	LCB desaturation	891.39928	IF	633.8894	IF	588.71726	IF
r403	LCB desaturation	744.41581	IF	858.1732	IF	898.85107	IF
r404	LCB hydroxylation	555.71359	IF	740.9661	IF	602.70542	IF
r405	LCB degradation	965.6077	IF	962.8005	IF	757.88787	IF
r406	LCB degradation	669.54014	IF	678.1669	IF	572.0321	IF
r407	LCB degradation	961.18543	IF	981.1921	IF	985.3995	IF
r408	LCB degradation	0.6124747	BF	0.886632	BF	0.6845704	BF
r409	LCB degradation	0.6124747	BF	0.886632	BF	0.6845704	BF
r410	Long-chain ceramide synthesis	2.7399016	BF	1.488821	BF	2.5018621	BF
r411	Long-chain ceramide synthesis	1.3103871	BF	1.71169	BF	1.9698516	BF
r412	Long-chain ceramide synthesis	1.7084488	BF	1.822309	BF	2.0970869	BF
r413	Long-chain ceramide synthesis	3.3413012	BF	2.752579	BF	2.0658496	BF
r414	Long-chain ceramide degradation	2.739888	BF	1.488661	BF	2.501841	BF
r415	Long-chain ceramide degradation	1.3095469	BF	1.70886	BF	1.9655798	BF
r416	Long-chain ceramide degradation	1.7077694	BF	1.821901	BF	2.097133	BF
r417	Long-chain ceramide degradation	3.3422485	BF	2.75117	BF	2.0645697	BF
r418	Very-long-chain ceramide synthesis	4.3578539	BF	7.715646	BF	4.3136348	BF
r419	Very-long-chain ceramide synthesis	8.7817641	BF	5.694421	BF	6.7077636	BF
r420	Very-long-chain ceramide synthesis	3.5295194	BF	2.408687	BF	2.9528709	BF
r421	Very-long-chain ceramide synthesis	4.2127446	BF	3.453244	BF	5.0346985	BF
r422	Very-long-chain ceramide synthesis	4.608139	BF	3.854737	BF	3.4107203	BF
r423	Very-long-chain ceramide synthesis	5.6709963	BF	7.263345	BF	6.1313472	BF
r424	Very-long-chain ceramide synthesis	3.5244325	BF	3.770128	BF	4.3695179	BF
r425	Very-long-chain ceramide synthesis	4.6239162	BF	3.720505	BF	3.7953091	BF
r426	Very-long-chain ceramide degradation	2.1905924	BF	4.899526	BF	2.6481642	BF
r427	Very-long-chain ceramide degradation	1.9294142	BF	1.400288	BF	2.3380799	BF
r428	Very-long-chain ceramide degradation	2.7824852	BF	2.919854	BF	2.5284588	BF
r429	Very-long-chain ceramide degradation	6.2532973	BF	3.182878	BF	2.3868717	BF
r430	Very-long-chain ceramide degradation	2.7924997	BF	3.175204	BF	3.2417941	BF
r431	Very-long-chain ceramide degradation	1.654875	BF	3.178278	BF	2.1331369	BF
r432	Very-long-chain ceramide degradation	3.7449578	BF	3.378223	BF	4.2554344	BF
r433	Very-long-chain ceramide degradation	2.7185461	BF	4.234617	BF	4.8273498	BF
r434	Ceramide LCB-hydroxylation	3.2847564	BF	3.234191	BF	4.0981726	BF
r435	Ceramide LCB-hydroxylation	2.3948574	BF	3.112839	BF	2.9876163	BF
r436	Long-chain ceramide alpha-hydroxylation	0.0002983	RF	0.000267	RF	0.0002828	RF
r437	Long-chain ceramide alpha-hydroxylation	0.0065064	RF	0.005832	RF	0.0061698	RF
r438	Long-chain ceramide alpha-hydroxylation	0.0006397	RF	0.000573	RF	0.0006066	RF
r439	Long-chain ceramide alpha-hydroxylation	0.0069564	RF	0.006235	RF	0.0065965	RF
r440	Very-long-chain ceramide alpha-hydroxylation	4.7098229	BF	3.792224	BF	3.5955032	BF
r441	Very-long-chain ceramide alpha-hydroxylation	9.5915915	BF	5.22986	BF	6.284673	BF
r442	Very-long-chain ceramide alpha-hydroxylation	0.0028754	RF	0.002577	RF	0.0027266	RF
r443	Very-long-chain ceramide alpha-hydroxylation	0.0023156	RF	0.002075	RF	0.0021958	RF
r444	Very-long-chain ceramide alpha-hydroxylation	4.7663799	BF	4.426039	BF	5.1332466	BF
r445	Very-long-chain ceramide alpha-hydroxylation	6.2567661	BF	5.436772	BF	6.3043047	BF
r446	Very-long-chain ceramide alpha-hydroxylation	2.916E-06	RF	2.61E-06	RF	2.765E-06	RF
r447	Very-long-chain ceramide alpha-hydroxylation	0.0126184	BF	0.01131	BF	0.0119656	BF
r448	Long-chain hydroxylceramide glucosylation	2.4344115	BF	2.286215	BF	4.6218412	BF
r449	Long-chain hydroxylceramide glucosylation	1.6341334	BF	2.627672	BF	1.7824886	BF
r450	Long-chain hydroxylceramide glucosylation	1.592099	BF	1.690888	BF	2.2631503	BF
r451	Long-chain hydroxylceramide glucosylation	1.9261375	BF	1.513117	BF	2.5673956	BF
r452	Long-chain glucosylceramide degradation	2.4344115	BF	2.286215	BF	4.6218412	BF
r453	Long-chain glucosylceramide degradation	1.634039	BF	2.627359	BF	1.7825829	BF
r454	Long-chain glucosylceramide degradation	1.5920983	BF	1.690883	BF	2.2631602	BF
r455	Long-chain glucosylceramide degradation	1.9267482	BF	1.513729	BF	2.568496	BF
r456	Very-long-chain hydroxylceramide glucosylation	3.118642	BF	2.280832	BF	1.7163731	BF
r457	Very-long-chain hydroxylceramide glucosylation	1.9581782	BF	3.500058	BF	2.1010147	BF
r458	Very-long-chain hydroxylceramide glucosylation	1.8737974	BF	2.168017	BF	1.6308077	BF
r459	Very-long-chain hydroxylceramide glucosylation	1.865647	BF	2.35413	BF	2.1378746	BF
r460	Very-long-chain hydroxylceramide glucosylation	2.2127127	BF	1.990514	BF	3.1107668	BF
r461	Very-long-chain hydroxylceramide glucosylation	1.9563111	BF	2.108021	BF	1.9282944	BF
r462	Very-long-chain hydroxylceramide glucosylation	2.773781	BF	2.214492	BF	2.2287123	BF
r463	Very-long-chain hydroxylceramide glucosylation	2.3197591	BF	2.983733	BF	4.8624845	BF
r464	Very-long-chain glucosylceramide degradation	3.1186404	BF	2.280831	BF	1.7163742	BF
r465	Very-long-chain glucosylceramide degradation	1.9582823	BF	3.49983	BF	2.1013099	BF
r466	Very-long-chain glucosylceramide degradation	1.8737974	BF	2.168017	BF	1.6308077	BF
r467	Very-long-chain glucosylceramide degradation	1.8649995	BF	2.353971	BF	2.1378732	BF
r468	Very-long-chain glucosylceramide degradation	2.2127127	BF	1.990514	BF	3.1107668	BF
r469	Very-long-chain glucosylceramide degradation	1.9565562	BF	2.107267	BF	1.9284395	BF
r470	Very-long-chain glucosylceramide degradation	2.773781	BF	2.214492	BF	2.2287123	BF
r471	Very-long-chain glucosylceramide degradation	2.3197341	BF	2.983678	BF	4.862662	BF

We used the criteria described by Oberhardt et al. (2010) to classify different reaction fluxes based on their flux ranges. RF represents Rigid Flux; IF represents Infinitely Flexible flux; BF represents Bounded Flexible flux.

## Discussion

Our FBA model and isotope labeling experiments systematically explored the alterations in the sphingolipid pathway that occur in response to SA and BTH. Traditional metabolic responses can cause significant changes in the concentrations of certain metabolites. However, the systematic responses caused by plant activators and phytohormones cannot be achieved by only doubling the concentration of certain nodes; these responses also affect the dynamic properties of the whole network. To detect the underlying changes of network parameters caused by the modulation, both up and down, of certain nodes, one of the most direct measurements is the fluxome. FBA analysis has been applied in microbial metabolic engineering and modeling of other systems. However, construction of the model for sphingolipid metabolism presented difficulties related to the unique features of sphingolipid pathways. Although sphingolipid species are among the most reactive components in plant development and stress responses, they reside in the periphery of the network of plant metabolism, having loose metabolic connections with other subnetworks. Their lack of connection and remote position make the flux in the self-balanced function more susceptible to the objective settings, rather than being affected by artificial constraints and neighboring subnetworks.

Indeed, studies of sphingolipids in *S. cerevisiae* (Ozbayraktar and Ulgen, 2011) found that the sphingolipid pathways are also remote from central metabolism, but these models are backed by experimental data on enzyme kinetic parameters or known fluxes. Experimental exploration of plant sphingolipid pathways has been hindered by the vast diversity, low abundance, and lack of sensitive and replicable measurements of sphingolipids. In addition, the enzymes linking metabolites often are embedded in the layers of membranes, making the isolation and estimation of their kinetic properties difficult. Until now, a limited set of experiments has revealed only a rough sketch of plant sphingolipid metabolism. Considering that, we used the experimentally measured isotopic incorporation rate to set the stoichiometry of each sphingolipid species in the objective function, and we found that the resulting flux distribution of each species was in accordance with the isotopic incorporation pattern, demonstrating that isotopic incorporation data produce a better fit than biomass fraction in objective stoichiometry determination, as the maximization of biomass is often considered as the aim of plant metabolism regardless of any inconsistency between biomass contents and the generation rate of each component.

In our experiments, isotopic transient labeling provided a direct measurement of *in vivo* flux. We note that none of the sphingolipid species reached 100% labeled. Similar phenomena were also observed in other experiments (Delwiche and Sharkey, 1993; Hasunuma et al., 2010). Considering the internal serine sources and anaplerotic reactions of complex existing sphingolipids, the pattern indicates a balance of labeled and unlabeled sphingolipids in the metabolic pool. Since the only exogenous source of nitrogen is labeled, we can also speculate that sphingolipid synthesis uses external and internal sources of nitrogen, based on the isotopic incorporation curve.

There are various models linking plant sphingolipid pathways with hormones and their synergistic roles in plant development and stress responses. In these models, the possible sphingolipid inducers of defense responses include LCBs (Saucedo-García et al., 2011) and ceramides (Markham et al., 2011; Bi et al., 2014), with SA both up- and downstream of sphingolipid-mediated PCD (Saucedo-García et al., 2011; Bi et al., 2014). As mutants affecting sphingolipids often accumulate SA, the effect of SA on ceramide species may include positive feedback on the imbalance of sphingolipids. Our results are in accordance with the observed frequent variation in the concentration of LCB and sometimes ceramide, and the reduced variation in the concentrations of hydroxyceramide and glucosylceramide in wild-type *Arabidopsis*. Functionally speaking, since LCB and ceramides are fundamental to sphingolipid metabolism and show high flexibility in their flux, they can be more responsive to stimuli such as SA or BTH without disrupting the total fluxomic balance of sphingolipid metabolism.

In a living cell, the synthesis and degradation of all substances occurs through metabolism. However, current research tends to separate metabolites and functional molecules. The most exciting aspect of plant sphingolipids is that they are metabolites and functional molecules. Our current model only deals with their metabolic properties in a self-balanced manner. It will be interesting to incorporate the signaling network that involves sphingolipids to build an integrated model that can consider the direct effect of metabolism on cellsignaling.

## Conclusion

In this study, we established a sphingolipid FBA model and used ^15^N-labeled isotopic transient labeling to systematically explore the effects of SA and BTH on sphingolipid metabolic pathways. The results show that increases in ceramide and glucosylceramide flux occur in response to exogenous SA and BTH and that alteration of their flux variability also occurs. Our results also give us insights that help explain the mechanism of crosstalk between SA and sphingolipids, and their roles in the plant defense response.

### Conflict of interest statement

The authors declare that the research was conducted in the absence of any commercial or financial relationships that could be construed as a potential conflict of interest.
