# Supplementary material for: A systematic simulation of the effect of salicylic acid on sphingolipid metabolism
Source: Front Plant Sci. 2015 Mar 25;6:186. doi: 10.3389/fpls.2015.00186 (PMC4373270; doi:10.3389/fpls.2015.00186)
Supplement: Supplementary file 1 [file Table1.DOCX]

**Table S1.** The indexes, categories, and equations of sphingolipid–related reactions in our FBA model.

| Index | Category | Equation | Source |
| --- | --- | --- | --- |
| r400 | LCB synthesis | c16:0_CoA + L_serine + NADPH + H -> d18:0_LCB + NADP + CoA + CO2 | AraCyc KEGG |
| r401 | LCB hydroxylation | d18:0_LCB + O2 + NADPH + H -> t18:0_LCB + NADP + H2O | KEGG |
| r402 | LCB desaturation | t18:0_LCB + NADH + H + O2 -> t18:1_LCB + NAD + 2 H2O | Sperling et al., 1998; Ryan et al., 2007 |
| r403 | LCB desaturation | d18:0_LCB + NADH + H + O2 -> d18:1_LCB + NAD + 2 H2O | Sperling et al., 1998; Ryan et al., 2007 |
| r404 | LCB hydroxylation | d18:1_LCB + O2 + NADPH + H -> t18:1_LCB + NADP + H2O | KEGG |
| r405 | LCB degradation | t18:1_LCB + ATP -> Hexadecanal + ADP + P + Ethanolamine | AraCyc KEGG |
| r406 | LCB degradation | d18:1_LCB + ATP -> Hexadecanal + ADP + P + Ethanolamine | AraCyc KEGG |
| r407 | LCB degradation | t18:0_LCB + ATP -> Hexadecanal + ADP + P + Ethanolamine | AraCyc KEGG |
| r408 | LCB degradation | d18:0_LCB + ATP -> Hexadecanal + ADP + P + Ethanolamine | AraCyc KEGG |
| r409 | LCB degradation | Hexadecanal + NAD + H2O -> c16:0 + NADH + H | AraCyc KEGG |
| r410 | ceramide synthesis | t18:0_LCB + c16:0_CoA + NADPH + H + O2 -> t18:0c16:0 + CoA + NADP + 2 H2O | AraCyc |
| r411 | ceramide synthesis | t18:1_LCB + c16:0_CoA + NADPH + H + O2 -> t18:1c16:0 + CoA + NADP + 2 H2O | AraCyc |
| r412 | ceramide synthesis | d18:0_LCB + c16:0_CoA + NADPH + H + O2 -> d18:0c16:0 + CoA + NADP + 2 H2O | AraCyc |
| r413 | ceramide synthesis | d18:1_LCB + c16:0_CoA + NADPH + H + O2 -> d18:1c16:0 + CoA + NADP + 2 H2O | AraCyc |
| r414 | ceramide degradation | t18:0c16:0 + H2O -> t18:0_LCB + c16:0 | KEGG |
| r415 | ceramide degradation | t18:1c16:0 + H2O -> t18:1_LCB + c16:0 | KEGG |
| r416 | ceramide degradation | d18:0c16:0 + H2O -> d18:0_LCB + c16:0 | KEGG |
| r417 | ceramide degradation | d18:1c16:0 + H2O -> d18:1_LCB + c16:0 | KEGG |
| r418 | ceramide synthesis | t18:0_LCB + c24:0_CoA + NADPH + H + O2 -> t18:0c24:0 + CoA + NADP + 2 H2O | AraCyc |
| r419 | ceramide synthesis | t18:1_LCB + c24:0_CoA + NADPH + H + O2 -> t18:1c24:0 + CoA + NADP + 2 H2O | AraCyc |
| r420 | ceramide synthesis | t18:0_LCB + c24:1_CoA + NADPH + H + O2 -> t18:0c24:1 + CoA + NADP + 2 H2O | AraCyc |
| r421 | ceramide synthesis | t18:1_LCB + c24:1_CoA + NADPH + H + O2 -> t18:1c24:1 + CoA + NADP + 2 H2O | AraCyc |
| r422 | ceramide synthesis | t18:0_LCB + c26:0_CoA + NADPH + H + O2 -> t18:0c26:0 + CoA + NADP + 2 H2O | AraCyc |
| r423 | ceramide synthesis | t18:1_LCB + c26:0_CoA + NADPH + H + O2 -> t18:1c26:0 + CoA + NADP + 2 H2O | AraCyc |
| r424 | ceramide synthesis | t18:0_LCB + c26:1_CoA + NADPH + H + O2 -> t18:0c26:1 + CoA + NADP + 2 H2O | AraCyc |
| r425 | ceramide synthesis | t18:1_LCB + c26:1_CoA + NADPH + H + O2 -> t18:1c26:1 + CoA + NADP + 2 H2O | AraCyc |
| r426 | ceramide degradation | t18:0c24:0 + H2O -> t18:0_LCB + c24:0 | KEGG |
| r427 | ceramide degradation | t18:1c24:0 + H2O -> t18:1_LCB + c24:0 | KEGG |
| r428 | ceramide degradation | t18:0c24:1 + H2O -> t18:0_LCB + c24:1 | KEGG |
| r429 | ceramide degradation | t18:1c24:1 + H2O -> t18:1_LCB + c24:1 | KEGG |
| r430 | ceramide degradation | t18:0c26:0 + H2O -> t18:0_LCB + c26:0 | KEGG |
| r431 | ceramide degradation | t18:1c26:0 + H2O -> t18:1_LCB + c26:0 | KEGG |
| r432 | ceramide degradation | t18:0c26:1 + H2O -> t18:0_LCB + c26:1 | KEGG |
| r433 | ceramide degradation | t18:1c26:1 + H2O -> t18:1_LCB + c26:1 | KEGG |
| r434 | ceramide LCB-hydroxylation | d18:0c16:0 + O2 + NADPH + H -> t18:0c16:0 + NADP + H2O | AraCyc KEGG |
| r435 | ceramide LCB-hydroxylation | d18:1c16:0 + O2 + NADPH + H -> t18:1c16:0 + NADP + H2O | AraCyc KEGG |
| r436 | ceramide alpha-hydroxylation | t18:0c16:0 + O2 + NADPH + H -> t18:0h16:0 + NADP + H2O | AraCyc |
| r437 | ceramide alpha-hydroxylation | t18:1c16:0 + O2 + NADPH + H -> t18:1h16:0 + NADP + H2O | AraCyc |
| r438 | ceramide alpha-hydroxylation | d18:0c16:0 + O2 + NADPH + H -> d18:0h16:0 + NADP + H2O | AraCyc |
| r439 | ceramide alpha-hydroxylation | d18:1c16:0 + O2 + NADPH + H -> d18:1h16:0 + NADP + H2O | AraCyc |
| r440 | ceramide alpha-hydroxylation | t18:0c24:0 + O2 + NADPH + H -> t18:0h24:0 + NADP + H2O | AraCyc |
| r441 | ceramide alpha-hydroxylation | t18:1c24:0 + O2 + NADPH + H -> t18:1h24:0 + NADP + H2O | AraCyc |
| r442 | ceramide alpha-hydroxylation | t18:0c24:1 + O2 + NADPH + H -> t18:0h24:1 + NADP + H2O | AraCyc |
| r443 | ceramide alpha-hydroxylation | t18:1c24:1 + O2 + NADPH + H -> t18:1h24:1 + NADP + H2O | AraCyc |
| r444 | ceramide alpha-hydroxylation | t18:0c26:0 + O2 + NADPH + H -> t18:0h26:0 + NADP + H2O | AraCyc |
| r445 | ceramide alpha-hydroxylation | t18:1c26:0 + O2 + NADPH + H -> t18:1h26:0 + NADP + H2O | AraCyc |
| r446 | ceramide alpha-hydroxylation | t18:0c26:1 + O2 + NADPH + H -> t18:0h26:1 + NADP + H2O | AraCyc |
| r447 | ceramide alpha-hydroxylation | t18:1c26:1 + O2 + NADPH + H -> t18:1h26:1 + NADP + H2O | AraCyc |
| r448 | hydroxyceramide glucosylation | t18:0h16:0 + UDP_glucose -> t18:0g16:0 + UDP | AraCyc |
| r449 | hydroxyceramide glucosylation | t18:1h16:0 + UDP_glucose -> t18:1g16:0 + UDP | AraCyc |
| r450 | hydroxyceramide glucosylation | d18:0h16:0 + UDP_glucose -> d18:0g16:0 + UDP | AraCyc |
| r451 | hydroxyceramide glucosylation | d18:1h16:0 + UDP_glucose -> d18:1g16:0 + UDP | AraCyc |
| r452 | glucosylceramide degradation | t18:0g16:0 + H2O -> t18:0h16:0 + glucose | KEGG |
| r453 | glucosylceramide degradation | t18:1g16:0 + H2O -> t18:1h16:0 + glucose | KEGG |
| r454 | glucosylceramide degradation | d18:0g16:0 + H2O -> d18:0h16:0 + glucose | KEGG |
| r455 | glucosylceramide degradation | d18:1g16:0 + H2O -> d18:1h16:0 + glucose | KEGG |
| r456 | hydroxyceramide glucosylation | t18:0h24:0 + UDP_glucose -> t18:0g24:0 + UDP | AraCyc |
| r457 | hydroxyceramide glucosylation | t18:1h24:0 + UDP_glucose -> t18:1g24:0 + UDP | AraCyc |
| r458 | hydroxyceramide glucosylation | t18:0h24:1 + UDP_glucose -> t18:0g24:1 + UDP | AraCyc |
| r459 | hydroxyceramide glucosylation | t18:1h24:1 + UDP_glucose -> t18:1g24:1 + UDP | AraCyc |
| r460 | hydroxyceramide glucosylation | t18:0h26:0 + UDP_glucose -> t18:0g26:0 + UDP | AraCyc |
| r461 | hydroxyceramide glucosylation | t18:1h26:0 + UDP_glucose -> t18:1g26:0 + UDP | AraCyc |
| r462 | hydroxyceramide glucosylation | t18:0h26:1 + UDP_glucose -> t18:0g26:1 + UDP | AraCyc |
| r463 | hydroxyceramide glucosylation | t18:1h26:1 + UDP_glucose -> t18:1g26:1 + UDP | AraCyc |
| r464 | glucosylceramide degradation | t18:0g24:0 + H2O -> t18:0h24:0 + glucose | KEGG |
| r465 | glucosylceramide degradation | t18:1g24:0 + H2O -> t18:1h24:0 + glucose | KEGG |
| r466 | glucosylceramide degradation | t18:0g24:1 + H2O -> t18:0h24:1 + glucose | KEGG |
| r467 | glucosylceramide degradation | t18:1g24:1 + H2O -> t18:1h24:1 + glucose | KEGG |
| r468 | glucosylceramide degradation | t18:0g26:0 + H2O -> t18:0h26:0 + glucose | KEGG |
| r469 | glucosylceramide degradation | t18:1g26:0 + H2O -> t18:1h26:0 + glucose | KEGG |
| r470 | glucosylceramide degradation | t18:0g26:1 + H2O -> t18:0h26:1 + glucose | KEGG |
| r471 | glucosylceramide degradation | t18:1g26:1 + H2O -> t18:1h26:1 + glucose | KEGG |
