# Supplementary material for: A systematic simulation of the effect of salicylic acid on sphingolipid metabolism
Source: Front Plant Sci. 2015 Mar 25;6:186. doi: 10.3389/fpls.2015.00186 (PMC4373270; doi:10.3389/fpls.2015.00186)
Supplement: Supplementary file 2 [file Table2.DOCX]

**Table S2.** Sphingolipid-related gene expression changes in SA- and BTH-treated plants from microarrays data published by van Leeuwen *et al.* (2007) and Wang *et al*. (2006). We picked genes that change more than 1.5-fold in SA-treated plants and 2-fold in BTH-treated plants. NA represents genes whose fold-changes were less than these thresholds. The fluxes corresponding to these genes were not adjusted during the simulation of SA and BTH effects in our FBA model.

| **TAIR ID** | **SA-treated** (van Leeuwen *et al.*, 2007) | **BTH-treated** (Wang *et al.*, 2006) | **Probe ID of GPL198** | **Gene symbol** | **Gene description** |
| --- | --- | --- | --- | --- | --- |
|  | **Log2(Fold change) of expression** | **Log2(Fold change) of expression** |  |  |  |
| At1g14290 | NA | -1.515 | 261492_at | SBH2 | Sphingoid base hydroxylase 2 |
| At2g46210 | 0.823 | NA | 266592_at | SLD2 | Fatty acid/sphingolipid desaturase 2 |
| At4g04930 | 0.678 | NA | 255276_at | SLDx | Sphingolipid delta-4 desaturase |
| At4g20870 | NA | -1.184 | 254448_at | FAH2 | Fatty acid hydroxylase 2 |
| At3g19260 | 1.111 | 1.104 | 257038_at | LOH2 | LAG one homologue 2 |
| At1g27980 | NA | 1.214 | 259598_at | AtDPL1 | Sphingosine-1-phosphate lyase |
